# Epidemiological characteristics of injury mortality in Guangdong Province, China, 2015

**DOI:** 10.1186/s12889-019-6437-6

**Published:** 2019-02-01

**Authors:** Ruilin Meng, Xiaojun Xu, Yanjun Xu, Chao Luo, Haofeng Xu, Ye Wang, Xiuling Song, Liang Xia, Ni Xiao, Shaoen Zhou, Lifeng Lin

**Affiliations:** 1 Institute of Control and Prevention for Chronic Non-infective Disease, Guangdong Provincial Center for Disease Control and Prevention, Guangzhou, China; 2 School of Public Health and Primary Care, The Chinese University of Hong Kong, Hong Kong, China; 3 Director’s office, Guangdong Provincial Center for Disease Control and Prevention, Center, Guangzhou, China

**Keywords:** Mortality, Injury, Prevention, Road-traffic accident

## Abstract

**Background:**

As the fourth leading cause of death, injury is an important public health concern in Guangdong Province, China. The epidemiological characteristics of injury mortality is changing along with the social development. This study described the epidemiological characteristics of injury mortality in Guangdong Province by analyzing the death surveillance data in a few areas in Guangdong Province in 2015.

**Methods:**

Using the mortality data from the Disease Surveillance Points (DSP) system, injury deaths were classified according to the International Classification of Disease-10th Revision (ICD-10). The data were stratified by areas (urban/rural), gender, age groups, injury types, and then overall and type-specific injury mortality rates were estimated for the whole Guangdong Province, China.

**Results:**

We estimated that about 38,200 individuals died from injury in Guangdong Province in 2015, producing a mortality rate of 43.11/100,000. The overall age-standardized injury mortality in men was higher in rural areas compared with urban areas (41.29/100,000 versus 24.89/100,000). In terms of injury intent, unintentional injuries were the commonnest injury type, which accounted for 83.93% of the overall injury deaths, however, the deaths caused by suicide should not be ignored, which occupied 12.67% of the total injury deaths. In terms of injury cause type, falls, road-traffic accidents, suicide, drowning, and accidental poisoning were the top five leading types of injury deaths.

**Conclusions:**

In Guangdong Province, injury is an important cause of death. Road-traffic accidents, falls, suicide, drowning, and accidental poisoning should be the priorities of intervention. Moreover, in rural areas, the men were the most targeted subpopulation of the prevention activities.

## Background

As a major public health problem, injury is responsible for a large proportion of the global disease burden. In 2015, about 5 million deaths were attributable to injury, which was equivalent to about 9% of total global mortality [1]. The pattern of injury mortality varies significantly across countries and areas along with the difference in essential demographic characteristics, economy income, customs, and lifestyle, etc. [2–8].

China has experienced dramatic changes in lifestyle, environment and economy (Gross Domestic Product (GDP) increased about 7% per year since 2012). All these changes have substantially resulted in changes in the injury mortality pattern [5, 6]. Injury-related mortality has decreased from 1990 to 2010, however the mortality of road-traffic injury is increasing in China. In 2010, the age-standardized injury mortality was 57/100,000 in China, with the leading causes of road-traffic injury, drowning, suicide and fall [5, 6]. The injury spectrums were different among provinces and areas according to the Disease Surveillance Points (DSP) system in China [7, 8]. The leading causes of injury death cis-position was suicide, road-traffic injury, drowning, falls in rural area, whereas, it was road-traffic injury, falls, suicide and drowning in urban areas of Hubei Province, China [7].

Guangdong Province, located in southern China, has a population of more than 100 million, making up 1/14 of the counry’s total population. The GDP of Guangdong Province is the first in the country. The injury was the fourth leading cause of deaths in Guangdong Province, which has drawn attention in recent years [9]. Along with the rapid social developments, the pattern of injury mortality might have changed greatly. However, the mortality patterns of injury in Guangdong Province remain unclear.

In the past 10 years, the number of surveillance areas in Guangdong province has gradually increased, with improved data quality as more resources have been allocated on the prevention and control of injuries. Therefore, we conducted this study using death data from the population-based mortality surveillance system to describe the epidemiological characteristics of injury mortality in Guangdong Province, China in 2015, and estimate the disease burden of injury in the whole province.

## Methods

### Data collection

The mortality data were collected through the population-based mortality surveillance system in Guangdong Province, China. This system was maintained by the Guangdong Provincial Center for Disease Control and Prevention (CDC). All deaths were reported and re-checked according to a quality control protocol as a part of routine processing operation at provincial, municipal and district levels in Guangdong Province. In 2015, Guangdong Provincial CDC collected the mortality data from 28 areas, 14 of which, including nine urban areas and five rural areas, were used in this analysis since they had a relatively higher data quality, with crude annual overall mortality rates higher than 5%.

### Classification of injury deaths

Injury causes were defined using the International Classification of Diseases, Tenth Revision (ICD–10). The codes identified the four major unintentional injury causes of death: transport injuries (V01-V04, V06, V09-V80, V87, V89 and V99), accidental poisonings (X40-X49), accidental falls (W00-W19), drowning (W65-W74), as well as other unintentional causes. In addition, the codes also identified two major intentional injury causes of death: self-inflicted injuries (X60-X84, Y87.0) and interpersonal violence (X85-Y09, and Y87.1), as well as legally interventions, war operations and other intentional causes [2, 10–12].

### Population

The number of people of all age groups (0, 1–4, 5–9, 10–14,... 85+), gender and areas (urban and rural) in the population were deduced according to the overall populations of Guangdong Province in 2015 issued by the Bureau of Statistics of Guangdong. The statistics of fifth and sixth population census and the age distribution of population released by the Center of Statistics and Information of National Health and Family Planning Commission.

### Data quality

The population size of the 14 areas was 9,959,716 (5,034,197 males and 4,925,519 females), accounting for 11.25% of the total population in Guangdong Province in 2015. Of them, 7,085,724 were from urban areas and 2,873,992 were from rural areas. In 2015, an under-reporting survey was conducted to investigate the completeness of the mortality data. Under-reporting rate is based on the total number of cases (M) in the surveillance and the estimated overall death toll (N) (under-reporting rate = (N-M) × 100%/N) [13]. The mortality rates were calculated according to the result of the under-reporting survey in 2015:

Adjusted mortality rate = crude mortality rate/ (1- under-reporting rate).

### Data analysis

We checked and evaluated data quality according to the under-reporting survey data quality control protocol and criteria. We combined and aggregated the data of 14 areas for the analysis. The mortality was adjusted according to the under-reporting rate. We calculated both crude rates (CR) and age-standardized rates (ASR) with the Chinese standard population and world standard population as standard population [14].

We estimated the numbers of injury deaths, age and sex-specific mortality for all the injury types from the 14 surveillance areas in Guangdong. For the most common types of injury (road-traffic injury, falls, drowning, suicide, poisoning, violence, and other types), the estimated numbers of injury deaths were stratified by urban/rural areas.

## Results

### Mortality rate

About 38,200 individuals died from injury in Guangdong Province in 2015. The majority of deaths occurred in rural areas (70.16%, 26,800/38,200), and in males (62.83%, 24,000/38,200). In 2015, the mortality of injury in the whole province was 43.11/100,000 (52.42/100,000 for males, 33.19/100,000 for females), with ASRc of 33.97/100,000 and ASRw of 34.39/100,000. The mortality rate of males were higher than that of females in both urban and rural areas. In urban areas, the injury mortality rate was 31.31/100,000 (38.93/100,000 for males, 23.49/100,000 for females), with ASRc of 24.01/100,000 and ASRw of 24.89/100,000. In rural areas, the injury mortality rate was 51.29/100,000 (61.52/100,000 for males, 40.14/100,000 for females), with ASRc of 41.21/100,000 and ASRw of 41.29/100,000. The injury mortality rate, ASRc and ASRw of both males and females in urban areas were lower than that in rural areas (Table 1).

**Table 1:** The injury mortality in Guangdong Province, 2015

Area	Gender	Deaths (× 10,000)	Mortality (1/10^5^)	ASMRC (1/10^5^)^a^	ASMRW (1/10^5^)^b^
All areas	Both	3.82	43.11	33.97	34.39
	Male	2.40	52.42	45.98	44.86
	Female	1.42	33.19	21.21	23.12
Urban	Both	1.14	31.31	24.01	24.89
	Male	0.72	38.93	33.17	33.09
	Female	0.42	23.49	14.8	16.6
Rural	Both	2.68	51.29	41.21	41.29
	Male	1.68	61.52	55.06	53.19
	Female	1.00	40.14	26.0	27.98

^a^Age standardized mortality rate (China population, 2000)

^b^Age standardized mortality rate (Segi’s population)

### Age-specific injury mortality rate

**Fig. 1:**
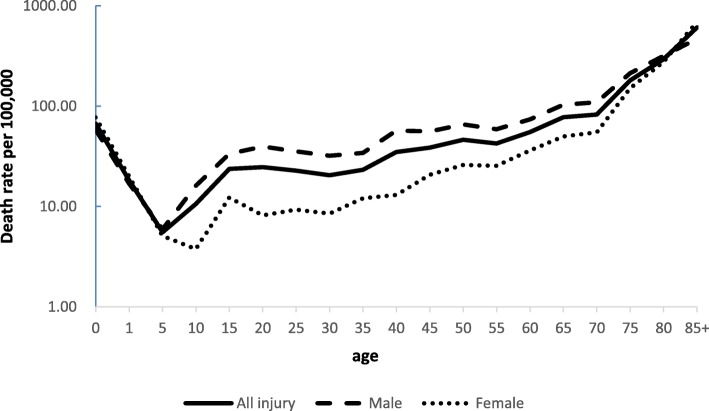
Injury death rates by sex in the single years of age: Guangdong Province, China, 2015

An approximate “U”-shaped association was observed between age and the injury mortality rate. The injury mortality rate gradually increased from the lowest in the 5-year age group until reaching the peak in over-85 age group. The same trend was also observed in males and females. The injury mortality rates of < 5 years and > 85 years was higher in females than that in males, however, the injury mortality rates of <=85 and > 5 years was lower in females than that in males (Fig. 1).

**Fig. 2:**
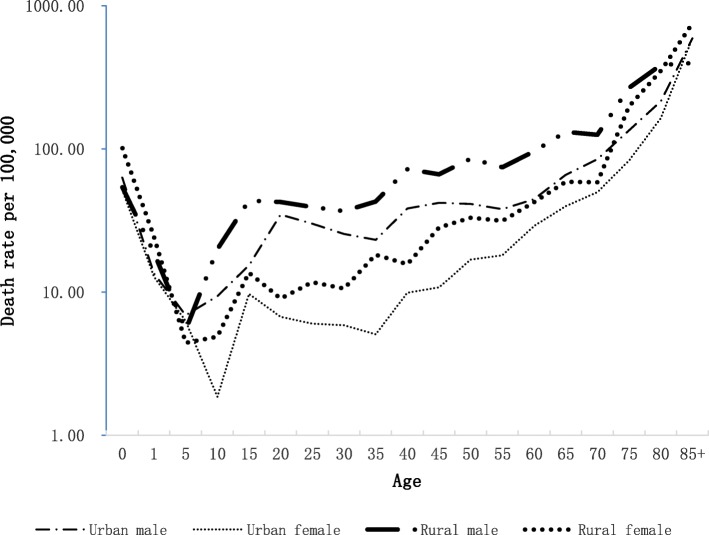
Injury death rates by sex in different areas by single years of age: Guangdong Province, China, 2015

The trends of the injury mortality rates in urban areas were similar to rural areas (Fig. 2). In comparison to urban areas, rural areas had higher injury mortality rates in all age groups except the 5-year age group. Dramatic rising in injury mortality rates was observed from 5-year to 20-year age group in males in both urban and rural areas. The injury mortality rates of both sex groups in urban and rural areas increased steadily after 20-year age group (Fig. 2, Table 2).

**Table 2:** The major injury mortality in Guangdong Province by gender and areas in 2015

		All areas			Urban			Rural	
Cause	Rate (1/10^5^)	ASR* (1/10^5^)	%	Rate (1/10^5^)	ASR* (1/10^5^)	%	Rate (1/10^5^)	ASR* (1/10^5^)	%
Both									
Unintentional	41.17	28.74	83.93	28.15	19.05	81.68	50.21	35.80	87.42
Fall	15.83	7.83	46.52	11.36	5.46	48.88	18.93	9.55	43.28
Transport accidents	13.56	11.55	37.46	8.41	7.18	36.21	17.14	14.76	39.18
Drowning	3.86	3.65	10.12	2.10	2.10	9.03	5.08	4.73	11.62
Poisoning	2.09	1.71	5.90	1.37	1.24	5.88	2.59	2.08	5.92
Intentional	6.13	4.54	13.52	4.99	3.81	14.47	6.92	5.05	12.06
Suicide	5.67	4.13	12.67	4.74	3.58	11.62	6.33	4.51	11.01
Homicide	0.44	0.39	0.78	0.21	0.19	0.57	0.60	0.54	1.04
Undetermined	0.72	0.56	2.55	1.33	1.04	3.85	0.30	0.19	0.52
Male									
Unintentional	49.96	40.18	83.89	33.82	26.44	80.24	60.84	49.95	89.64
Fall	14.63	9.55	35.99	10.99	6.77	38.78	17.09	11.58	32.23
Transport accidents	20.39	17.69	46.01	12.53	10.92	44.22	25.68	22.45	48.43
Drowning	5.14	5.08	11.03	2.78	2.95	9.80	6.74	6.52	12.71
Poisoning	2.92	2.33	6.96	2.04	1.76	7.21	3.52	2.76	6.63
Intentional	6.67	5.07	13.53	6.71	5.42	15.92	6.64	4.83	9.78
Suicide	6.18	4.63	12.70	6.36	5.08	15.10	6.05	4.31	8.92
Homicide	0.46	0.41	0.73	0.27	0.27	0.64	0.59	0.52	0.86
Undetermined	0.89	0.73	2.57	1.62	1.32	3.84	0.39	0.28	0.58
Female									
Unintentional	32.20	16.80	83.98	22.49	11.76	83.93	39.15	20.58	84.06
Fall	17.04	5.85	63.20	11.72	4.13	64.62	20.85	7.04	61.19
Transport accidents	6.60	5.31	23.92	4.31	3.53	23.73	8.24	6.73	24.18
Drowning	2.55	2.12	8.67	1.42	1.20	7.84	3.36	2.76	9.85
Poisoning	1.24	1.10	4.21	0.69	0.75	3.81	1.63	1.40	4.78
Intentional	5.57	4.06	13.51	3.27	2.28	12.20	7.22	5.37	15.50
Suicide	5.15	3.68	12.64	3.11	2.17	11.62	6.61	4.80	14.19
Homicide	0.42	0.39	0.87	0.15	0.12	0.57	0.61	0.56	1.31
Undetermined	0.55	0.39	2.51	1.04	0.77	3.87	0.20	0.11	0.44

* age standardized mortality rate (China population 2000)

### Intent and mechanism of injury

#### Injury intents

In 2015, 83.93% of injury deaths was classified as unintentional injuries (mortality rate of 41.17/100,000, ASRc of 28.74/100,000), 12.67% as suicide (mortality rate of 5.67/100,000, ASRc of 4.13/100,000), 2.55% as of undetermined intents (mortality rate of 0.72/100,000, ASRc of 0.56/100,000), 0.78% as due to violence (mortality rate of 0.44/100,000, ASRc of 0.39/100,000). The intents-of-injury by sex and area were distributed in a pattern similar to the overall intents-of-injury in the whole province (Table 2).

**Fig. 3:**
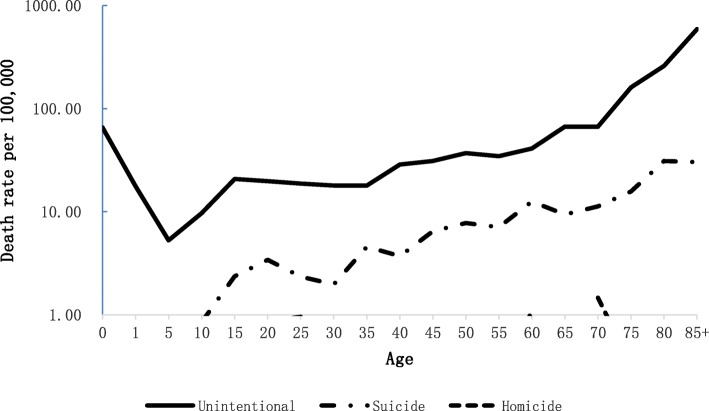
Injury death rates by intent of injury and single years of age: Guangdong Province, China, 2015

The unintentional injury mortality rate bottomed out in the 5-year age group and then gradually increased till peaking in the over 85 years of age group. The suicide mortality rate consistently grew with slight fluctuations since suicide firstly observed at the age of 10. The violence-related mortality rates were lower than 1/100,000 in most age groups except that in 60 years and 70 years of age group (Fig. 3).

#### Mortality spectrum of unintentional injuries

Overall, the first leading cause of injury deaths was accidental falls (with a mortality rate of 15.83/100,000), followed by road traffic accidents, drowning and accidental poisoning, with mortality rates of 13.56, 3.86 and 2.09 per 100,000 respectively. For males, the four leading causes of injury deaths were road traffic accidents (mortality rate of 20.39/100,000), falls (mortality rate of 14.63/100,000), drowning (mortality rate of 5.14/100,000) and accidental poisoning (mortality rates of 2.92/100,000). For females, falls was the leading mechanism of injury deaths with of mortality rate of 17.04/100,000, followed by road traffic accidents, drowning and accidental poisoning, with mortality rates of 6.60, 2.55 and 1.24 per 100,000 respectively (Table 2).

Similar patterns of the leading causes of injury deaths were observed in the subgroups stratified by sex and area. Falls, road traffic accidents, drowning and accidental poisoning were still the four leading mechanisms of injuries deaths in urban and rural areas. However, the injury mortality rates of these four mechanisms in rural areas were almost twice of that in urban areas. The mortality rates of falls, road traffic accidents, drowning and accidental poisoning were 11.36, 8.41, 2.10 and 1.37 per 100,000 in urban areas; 18.93, 17.14, 5.08 and 2.59 per 100,000 in rural areas respectively (Table 2).

As the first leading mechanism of injury deaths in males, the mortality rates of road traffic accidents were 12.53/100,000 in urban areas and 25.68/100,000 in rural areas. Falls were the second leading mechanism of injury deaths in males. The mortality rates of falls were 10.99/100,000 in urban males and 17.09/100,000 in rural males. The third and fourth leading mechanisms of injury deaths were drowning and accidental poisoning. The mortality rates of drowning were 2.78 and 6.74 per 100,000 in males of urban and rural areas; the mortality rates of accidental poisoning were 2.04 and 3.52 per 100,000 in urban and rural areas (Table 2).

As the first leading mechanism of injury deaths in females, the mortality rates of falls were 11.72/100,000 in urban females, 20.85/100,000 in rural females. Road traffic accidents were the second leading mechanism of injury deaths in females. The mortality rates of road traffic accidents were 4.31/100,000 in urban areas and 8.24/100,000 in rural areas. The third and fourth leading mechanisms of injury deaths were drowning and accidental poisoning. The mortality rates of drowning were 1.42 and 3.36 per 100,000 in urban and rural areas; the mortality rates of accidental poisoning were 0.69 and 1.63 per 100,000 in females of urban and rural areas. (Table 2).

## Discussion

In the current study, we provided a comprehensive description of the injury mortality pattern using the latest and most representative data in Guangdong Province, China. The results showed that injury is an important public health problem in Guangdong Province. We summarized the epidemiological characteristics as follows: 1) Crude and age-adjusted injury mortality rates were significantly higher in rural areas than in urban areas, and higher in males than in females. 2) The leading cause of injury deaths was unintentional injuries, but suicide should not be ignored. 3) The mortality spectrum of unintentional injuries was different between rural and urban areas, males and females.

The injury mortality rate in Guangdong Province was lower than that of the global average and national average [11, 15]. The followings may explain the results: there were abundant health resources in Guangdong Province with higher economy than the national average. In addition, there were sufficient emergency medical services and higher awareness of disease prevention in Guangdong Province.

In comparison to the Third National Retrospective Survey on Causes of Death in 2004–2005 [16, 17], the injury mortality rate had decreased in Guangdong Province, with similar tendency to that of the national and other areas [5, 7, 11, 18]. In the past decade, the declining trend of injury mortality may be due to the enforcement of the Traffic Safety Law which provides punishments for both “driving while intoxicated” and “driving after drinking alcohol” in China [19–23].

The mortality spectrum of injuries in Guangdong Province was similar to the global and national situation [11, 15], the majority of injury deaths was due to unintentional injuries, which constituted 83.93% of all injury deaths in Guangdong Province in 2015. At the same time, suicide could not be ignored, which consisted of 12.67% of all injury deaths. Compared with the global and national suicide mortality rates, the mortality rate of suicide was relatively lower in Guangdong Province [11, 15, 24, 25]. However, homicide was one of the leading causes of injury deaths in other countries [4, 26, 27]. In comparison to the 3rd NRSCD, the first leading causes of injury changed from road traffic accidents to falls, suicide became third leading cause of injury. Therefore, we should pay more attention to falls while Guangdong Province enters an aging society.

The top four leading causes of injury deaths (falls, road traffic accidents, suicide, drowning) in Guangdong Province were different from the whole world (road traffic accidents, suicide, falls and violence) [15], the whole country (road-traffic accidents, falls, drowning, accidental poisoning) [11, 15, 28] and South Africa (homicide, road traffic injuries, suicide and fires, burns and hot substances) [4]. One important underlying reason for the high falls mortality rate was aging, we suggest that Guangdong Province should implement more appropriate strategies (the need for improvement of environmental conditions in communities and home) to prevent the occurrence of falls.

In Guangdong Province, crude and age-adjusted injury mortality rates in 2015 were higher in rural areas and in males. It is consistent with other reports in China [7, 29–31], and developed countries, such as Australia, Canada [32, 33] and Norway [34]. Generally, individuals in rural areas, are more likely to engage in higher risk work than in urban areas, males were also in higher risk-taking behaviors than females. Limited medical resources and lower levels of health care may also explain the above results. More government-funded health resources and services should be allocated to the injury control in rural areas in Guangdong Province to reduce these apparent urban and rural inequalities.

The injuries mortality rates of road traffic accidents, drowning and accidental poisoning in rural areas were twice of that in urban areas. Similar findings had been reported elsewhere in China [7, 29, 30]. The mortality rate in males was three times higher than that in females in both urban and rural areas. The falls-related injury mortality rates higher in females, whereas, the injury mortality rates resulting from road traffic accidents, drowning and accidental poisoning were higher in males.

These results will serve as a baseline for future assessment of the overall effectiveness of the injury control efforts in Guangdong Province and will provide insights into the areas of greatest need for prioritization.

A few limitations should be noted. Firstly, the current study was based on the surveillance data in a single year, the injury mortality rates lack longitudinal applicability. Secondly, along with the urbanization, development is evident in the rural areas, including the extension of health services and the change of production structure. Growth and development in the urban and rural areas of Guangdong Province are likely to influence the rate and type of injuries over time. Thirdly, the data from the 14 surveillance areas were composed of nine urban areas and five rural areas, with more population in urban areas than in rural areas.

## Conclusion

Injury is an important cause of death. Road-traffic accidents, falls, sucide, drowning, and accidental poisoning should be the priorities of intervention. Moreover, in rural areas, men were the most targeted subpopulation of the prevention activities.

## Abbreviations

ASRc: Age-standardized rates by the China population
ASRw: Age-standardized rates by the world population
CDC: Center for Disease Control and Prevention
ICD: International Classification of Diseases
WHO: World Health Organization

## References

[1] WHO. The top 10 causes of death. https://www.who.int/mediacentre/factsheets/fs310/en/index1.html. Accessed 24 May 2018.

[2] Bergen G, Chen LH, Warner M, Fingerhut LA (2008). Injury in the United States: 2007 Chartbook.

[3] Burrows S, Auger N, Lo E (2015). Language and unintentional injury mortality in Quebec. Canada Inj Prev.

[4] Richard M, Megan P, Victoria PW, Nomonde G, Shanaaz M, Lorna JM (2015). Injury-related mortality in South Africa: a retrospective descriptive study of postmortem investigations. Bull World Health Organ.

[5] Ning PS, Cheng XJ, Zhang L, Zhang W, Hu GQ (2015). Injury mortality in China, from 1990 to 2010. Zhonghua Liu Xing Bing Xue Za Zhi.

[6] Wang LJ, Liu YN, Liu SW, Yin P, Liu JM, Zeng XY (2015). Status injury burden in 1990 and 2010 for Chinese people. Zhonghua Yu Fang Yi Xue Za Zhi.

[7] Liu Q, Zhang L, Li JL, Zuo D, Kong DG, Shen XF (2012). The gap in injury mortality rates between urban and rural residents of Hubei province, China. BMC Public Health.

[8] Lin YL, Chen M, Chen GW, Wu XQ, Lin TQ (2015). Application of an autoregressive integrated moving average model for predicting injury mortality in Xiamen, China. BMJ Open.

[9] Xie HY, Xu XJ, Ma WJ, Tan HR, Zhong AM, Xu YJ (2011). Pattern of injury deaths among residents in Guangdong Province, 2004-2005. South China J Prev Med.

[10] World Health Organization. WHO Family of International Classifications, edit. The international statistical classification of diseases and related health problems 10th revision (ICD-10). Beijing: People’s Medical Publishing House; 2010. p. 789–876.

[11] Chinese Centre for Disease Control and Prevention. National disease surveillance system-Death cause surveillance dataset, 2015. Beijing: Science and Technology of China Press; 2016. p. 60–62.

[12] Miniño AM, Anderson RN, Fingerhut LA, Boudreault MA, Warner M (2006). Deaths: Injuries, 2002. Natl Vital Stat Rep.

[13] Kang G, Yin P, Wang LJ, Ji YB, Li QF, David B (2015). Propensity score weighting for addressing under-reporting in mortality surveillance: a proof-of-concept study using the nationally representative mortality data in China. Popul Health Metrics.

[14] Omar BA, Cynthia BP, Alan DL, Christopher JLM, Rafael L, Mie I. Age standardization of rates: a new WHO standard (technical report). GPE discussion paper series. 2001; no.31. World Health Organization (WHO).

[15] Haagsma JA, Graetz N, Bolliger I, Naghavi M, Higashi H, Mullany EC (2016). The global burden of injury: incidence, mortality, disability-adjusted life years and time trends from the global burden of disease study 2013. Inj Prev.

[16] Ma WJ (2010). Study on the injury spectrum, disease burden and related risk behaviors of residents in Guangdong Province.

[17] Chen Z (2008). The third national retrospective sampling survey of death cause report.

[18] Zhu YC, Jiang X, Li H, Wang Y, Xu GZ (2016). Demographic factors associated with leading causes of injury mortality in Ningbo, China: 2004-2013. Asia Pac J Public Health.

[19] Krauss EM, Dyer DM, Laupland KB, Buckley R (2010). Ten years of all-terrain vehicle injury, mortality, and healthcare costs. J Trauma.

[20] Huang QL, Liu F, Zhang J, Hu YH (2016). Analysis on the situation of road traffic safety and injury in Suzhou city, 2011-2014. Chinese J Health Educ.

[21] Taylor BJ, Shield KD, Rehm JT (2011). Combining best evidence: a novel method to calculate the alcohol-attributable fraction and its variance for injury mortality. BMC Public Health.

[22] Ning PS, Schwebel DC, Huang HL, Li L, Li J, Hu GQ (2016). Global Progress in road injury mortality since 2010. PLoS One.

[23] Xie SH, Wu YS, Liu XJ, Fu YB, Li SS, Ma HW (2016). Mortality from road traffic accidents in a rapidly urbanizing Chinese city: a 20-year analysis in Shenzhen, 1994-2013. Traffic Inj Prev.

[24] Kolves K, Potts B, De LD (2015). Ten years of suicide mortality in Australia: socio-economic and psychiatric factors in Queensland. J Forensic Legal Med.

[25] Rockett IRH, Lilly CL, Jia HM, Larkin GL, Miller TR, Nelson LS (2016). Self-injury mortality in the United States in the early 21st century: a comparison with proximally ranked diseases. JAMA Psychiatry.

[26] Rockett IRH, Regier MD, Kapusta ND, Jeffrey HC, Ted RM, Randy LH (2012). Leading causes of unintentional and intentional injury mortality: United States, 2000–2009. American J of Public Health.

[27] Prinsloo M, Matzopoulos R, Laubscher R, Myers J, Bradshaw D (2016). Validating homicide rates in the Western Cape Province, South Africa: findings from the 2009 injury mortality survey. S Afr Med J.

[28] Kim WC, Byiringiro JC, Ntakiyiruta G, Kyamanywa P, Irakiza JJ, Mvukiyehe JP (2016). Vital statistics: estimating injury mortality in Kigali, Rwanda. World J Surg.

[29] Jiang G, Choi BC, Wang D, Zhang H, Zheng W, Wu T (2009). Leading causes of death from injury and poisoning by age, sex and urban/rural areas in Tianjin, China 1999-2006. Injury.

[30] Wang SY, Li YH, Chi GB, Xiao SY, Ozanne SJ, Stevenson M (2008). Injury-related fatalities in China: an under-recognised public-health problem. Lancet.

[31] Hu GQ, Baker SP, Baker TD (2010). Urban-rural disparities in injury mortality in China, 2006. J Rural Health.

[32] Mitchell RJ, Chong SL (2010). Comparison of injury-related hospitalised morbidity and mortality in urban and rural areas in Australia. Rural Remote Health.

[33] Lagace C, Desmeules M, Pong RW, Heng D (2007). Non-communicable disease and injury-related mortality in rural and urban places of residence: a comparison between Canada and Australia. Can J Public Health.

[34] Kristiansen T, Rehn M, Gravseth HM, Lossius HM, Kristensen P (2012). Paediatric trauma mortality in Norway: a population-based study of injury characteristics and urban-rural differences. Injury.

